# Development and Validation of a Stability-Indicating RP-HPLC Method for the Simultaneous Determination of Phenoxyethanol, Methylparaben, Propylparaben, Mometasone Furoate, and Tazarotene in Topical Pharmaceutical Dosage Formulation

**DOI:** 10.3797/scipharm.1303-22

**Published:** 2013-06-04

**Authors:** Chinmoy Roy, Jitamanyu Chakrabarty

**Affiliations:** 1 Analytical Research and Development, Integrated Product Development, Dr. Reddy’s Laboratories Ltd., Bachupally, Hyderabad-500090, Andhra Pradesh, India.; 2 Department of chemistry, National Institute of Technology, Durgapur-713209, West Bengal, India.

**Keywords:** Mometasone Furoate, Tazarotene, Phenoxyethanol, Parabens, Chromatography, Degradation, RP-HPLC

## Abstract

A stability-indicating RP-HPLC method has been developed and validated for the simultaneous determination of phenoxyethanol (PE), methylparaben (MP), propylparaben (PP), mometasone furoate (MF), and tazarotene (TA) in topical pharmaceutical dosage formulation. The desired chromatographic separation was achieved on the Waters X-Bridge™ C18 (50×4.6mm, 3.5μ) column using gradient elution at 256 nm detection wavelength. The optimized mobile phase consisted of 0.1%v/v orthophosphoric acid in water as solvent-A and acetonitrile as solvent-B. The method showed linearity over the range of 5.88–61.76 μg/mL, 0.18–62.36 μg/mL, 0.17–6.26 μg/mL, 0.47–31.22 μg/mL, and 0.44–30.45 μg/mL for PE, MP, PP, MF, and TA, respectively. The recovery for all of the components was in the range of 98–102%. The stability-indicating capability of the developed method was established by analysing the forced degradation samples, in which the spectral purity of PE, MP, PP, MF, and TA along with the separation of degradation products from the analyte peaks was achieved. The proposed method was successfully applied for the quantitative determination of PE, MP, PP, MF, and TA in a cream sample.

## Introduction

**Fig. 1:**
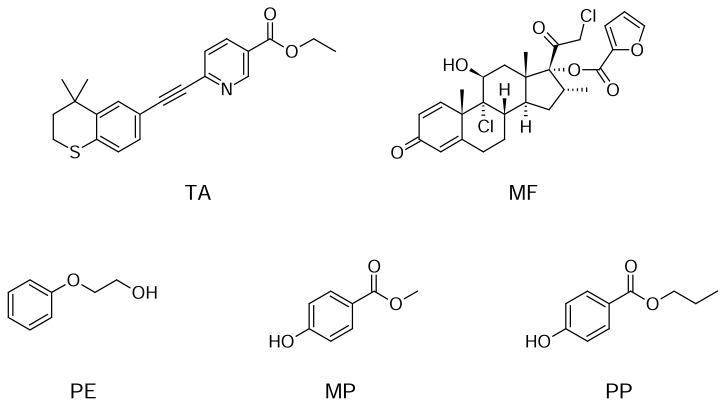
Chemical Structure of TA, MF, PE, MP, and PP.

Mometasone furoate (MF), (11β,16α)-9,21-dichloro-11-hydroxy-16-methyl-3,20-dioxo-pregna-1,4-dien-17-yl furan-2-carboxylate (Fig. 1), is a topical corticosteroid; it has anti-inflammatory, anti-pruritic, and vasoconstrictive properties. Mometasone inhibits the action of allergic reactions, eczema, and psoriasis that cause inflammation, redness, and swelling [1, 2].

Tazarotene (TA), ethyl 6-[(4,4-dimethyl-3,4-dihydro-2*H*-thiochromen-6-yl)ethynyl] nicotin-ate (Fig. 1), is a member of a new generation of receptor-selective, synthetic retinoids for the topical treatment of mild to moderate plaque psoriasis, acne vulgaris, and photoaging [3–5]. Psoriasis is one of the most common human skin diseases and is characterized by excessive growth and aberrant differentiation of corneocytes, but is fully reversible with appropriate therapy [6–8].

TA in combination with a mid-potency topical corticosteroid like MF is a valuable first-line treatment option for stable plaque psoriasis. Concurrent use of retinoids and steroids also enhances the speed of efficacy, patient satisfaction, and tolerability [9–11].

The preservative system is an important part of semisolid formulations in preventing the deterioration of formulations from microbial contamination. Methylparaben (MP), propylparaben (PP), and their salts are the most commonly used preservatives and have been used for many years. To establish their effectiveness throughout the shelf life of the product, the actual concentrations of preservatives must be determined, as also required by regulatory agencies [12, 13].

Phenoxyethanol (PE) is a colourless non-allergenic oily liquid that acts as a bactericide and also reduces the need for other preservatives by 10–20-fold.

The finished product released and the shelf life specifications should include an identification test and a content determination test with acceptance criteria and limits for each antimicrobial preservative present in the formulation [14–15]. Hence, their (PE, MP, and PP) antimicrobial and antifungal properties make them an integral part of the product formulation. This encourages the development of a new stability-indicating method for the simultaneous estimation of all the compounds (PE, MP, PP, MF, and TA) to provide the driving force in today’s pharmaceutical industry.

A detailed literature survey for PE, MP, PP, MF, and TA revealed that the determination of each individual compound or in combination with other drugs has been reported using HPLC [1, 2, 11, 13, 16–31], LC-MS [32, 33], electrophoresis [34], and spectrophotometric techniques [35].

The combination of MF and TA is not official in any pharmacopoeia. So far, no reversed-phase liquid chromatography (RPLC) stability-indicating method has been reported for the rapid and simultaneous determination of PE, MP, PP, MF, and TA in topical pharmaceutical formulation. Therefore, it is necessary to develop a new rapid and stability-indicating method for the simultaneous determination of five compounds (PE, MP, PP, MF, and TA) in topical pharmaceutical formulation. The proposed method is able to separate PE, MP, PP, MF, and TA from each other and also from the other degradation products. Furthermore, this method was validated according to ICH guidelines [37] and successfully applied for the separation and quantification of all compounds of interest in the topical pharmaceutical formulation. The chemical structures for all of the compounds are presented in Figure 1.

## Results and Discussion

### Method Development and Optimization

The primary target of the developed HPLC method is to achieve the simultaneous determination of PE, MP, PP, MF, and TA in topical formulations under common chromatographic conditions; those that are applicable to routine quality control of the products in the pharmaceutical and cosmetic industries.

The optimization of column selection and mobile phase selection were done simultaneously. An isocratic method was employed using a buffer (0.02M ammonium acetate pH 2.5 with glacial acetic acid), acetonitrile, and methanol in the ratio of 50:25:25 v/v/v, respectively, as the mobile phase. The X-Terra™ C18 (50×4.6mm, 5μ) column with a flow rate 1.5 mL/min at column temperature 40°C was used in the HPLC, equipped with a photodiode array detector. TA peak fronting was observed and the peak was eluted too late. To reduce the run time and improve the TA peak shape, an attempt was made by replacing methanol with acetonitrile from the mobile phase component which then became 0.02M ammonium acetate (pH 2.5 with glacial acetic acid) and acetonitrile in the ratio of 50:50 v/v. The column was changed to the Waters X-Bridge™ C18 (50×4.6mm, 3.5μ) for better peak shape. The TA peak eluted at 8.0 minutes but the PE and MP peaks were co-eluting in same retention time in the column void. To separate PE from the MP peak, an attempt was made with gradient elution with the mobile phase (0.02M ammonium acetate pH 2.5 adjusted with glacial acetic acid) as solvent-A and acetonitrile as solvent-B. PE was separated from the MP peak, and also the peak tailing 1.0 was observed for TA. The peak shapes for all of the components were good, but blank interference was observed at the retention time of MF. To remove the blank interference at the retention time of MF, solvent-A was changed to a 0.1%v/v orthophosphoric acid buffer, while keeping acetonitrile as solvent-B with the same gradient mode. As a result, no blank interference was observed. But when the base degradant sample was injected, the MF peak was eluted along with the base degradant peak. To separate the MF peak from the base degradant peak, the gradient programme was modified as time (min)/mobile phase-A (%)/mobile phase-B (%); 0.0/90/10, 1.5/90/10, 4.5/78/22, 8/50/50, 10.5/50/50, 15/5/95, 17/90/10, 20/90/10. While the flow rate was 1.5 mL/min and the column temperature was 50°C, the MF peak was separated from the base degradant peak. Good peak shape for all of the components with well-resolved degradant peaks were observed. Also, the resolution between the PE and MP peak was greater than 2.7. The wavelength was selected by injecting a known concentration of each of PE, MP, PP, MF, and TA into the HPLC with a PDA detector, and was evaluated for the UV spectra of each component. A common wavelength for the simultaneous determination of all the components was selected as 256 nm by overlaying the spectra and wavelength at which all components had significant absorbance.

**Fig. 2:**
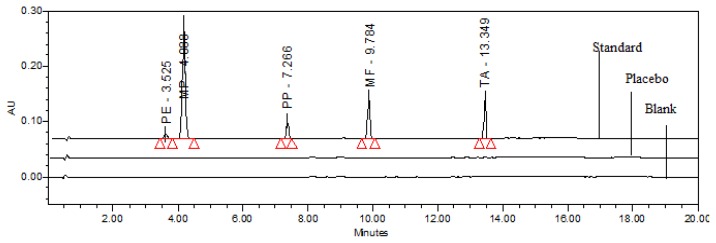
Typical overlay chromatogram of blank and placebo and standard preparation

The extraction of the active components from a semisolid sample matrix with acceptable recovery is a very critical aspect for sample preparation and was achieved by selecting the right diluent in the following manner. Considering the solubility of all the components, the mixture of acetonitrile and water in the ratio of 80:20 (v/v) was used as the diluent and satisfactory recovery was achieved. Based on the above experimental data, the chromatographic separation was finalized by following gradient program time (min)/mobile phase-A (%)/mobile phase-B (%); 0.0/90/10, 1.5/90/10, 4.5/78/22, 8/50/50, 10.5/50/50, 15/5/95, 17/90/10, 20/90/10, at a flow rate of 1.5 mL/min at 50°C (column oven) temperature, detection wavelength 256 nm with 10 μL injection volume. By using the above chromatographic conditions and diluent; the standard, sample, and placebo preparation were made and injected into the HPLC with the developed parameters (Fig. 2).

### Analytical Method Validation

After satisfactory development of the method, it was subjected to method validation as per ICH guidelines [36, 37]. The method was validated to demonstrate that it is suitable for its intended purpose by the standard procedure to evaluate adequate validation characteristics (system suitability, accuracy, precision, linearity, limit of detection, limit of quantification, robustness, solution stability, filter compatibility, and stability-indicating capability).

### System Suitability

System suitability parameters were measured so as to verify the system, method, and column performance. The system precision was determined by five replicate injections of the standard preparation. Results of the system suitability parameters such as % RSD, theoretical plates, and tailing factor are presented in Table 1.

### Method Precision (Repeatability)

The precision of the assay method was evaluated by carrying out six independent determinations of 40 μg/mL of PE, 40 μg/mL of MP, 4 μg/mL of PP, 20 μg/mL of MF, and 20 μg/mL of TA in cream samples against qualified working standards. The average % assay (n=6) of PE, MP, PP, MF, and TA were 100.6%, 101.4%,101.8%, 101.5%, and 98.6%, respectively, with the RSD below 0.7%. Low values of the % RSD indicate that the method is precise (Table 2).

### Intermediate Precision (Reproducibility)

The purpose of this study is to demonstrate the reliability of the test results with variations. The reproducibility was checked by analyzing the samples by a different analyst using a different chromatographic system and column on a different day. Results are presented in Table 2.

### Specificity

Specificity is the ability of the method to measure the analyte response in the presence of its potential impurities and placebo matrix [37]. Forced degradation studies were performed to demonstrate the selectivity and stability-indicating capability of the proposed RP-LC method. Figure 2 shows that there is no interference at the retention time of PE, MP, PP, MF, and TA due to the blank or placebo. Overlay chromatograms of the blank, placebo, and standard are presented in Figure 2.

### Forced Degradation Studies

Force degradation studies of the drug product were also performed to evaluate the stability-indicating property and specificity of proposed method. Stress studies were performed at the concentration of 40 μg/mL of PE, 40 μg/mL of MP, 4 μg/mL of PP, 20 μg/mL of MF, and 20 μg/mL of TA on the cream formulation. The peak purity test was carried out for the PE, MP, PP, MF, and TA peaks by using a PDA detector on the stress samples. All the solutions used in the forced degradation studies were prepared by dissolving the drug product in a small volume of diluent and further stressing agents. After degradation, these solutions were diluted with diluent to yield the stated PE, MP, PP, MF, and TA concentrations of 40 μg/mL, 40 μg/mL, 4 μg/mL, 20 μg/mL, and 20 μg/mL, respectively.

#### Acid Hydrolysis

Acidic degradation was carried out by adding 1 mL of 0.1N HCl, and after 45 minutes neutralizing the mixture by adding 1 mL 0.1N NaOH. Fig 5 (a) shows significant degradation was observed for MF and one major degradation peak was observed at 10.039 min. Degradation was also observed for TA with a degradation peak at 6.323 min. All the major and minor degradation products were well-separated from the PE, MP, PP, MF, and TA peaks. The peak purity was checked for all five analytes and the results are summarized in Table 3.

#### Base Hydrolysis

Basic degradation was carried out by adding 0.5 mL of 0.05N NaOH, and after 15 minutes neutralizing the mixture by adding 0.5 mL 0.05 HCl. Fig 5 (b) shows significant degradation was observed for MF and one major degradation peak was observed at 10.036 min. All the degradation products were well-separated from the PE, MP, PP, MF, and TA peaks. The peak purity was checked for all five analytes and the results are summarized in Table 3.

#### Hydrogen Peroxide Oxidation

Peroxide oxidation was carried out by adding 1 mL of 30%v/v H_2_O_2_, at 70°C for 30 minutes. Fig 5 (c) shows significant degradation for TA was observed when the cream sample was subjected to peroxide oxidation and one main degradation peak was observed at 8.443 min. All the degradation products were well-separated from the PE, MP, PP, MF, and TA peaks. The peak purity was checked for all five analytes and the results are summarized in Table 3.

#### Thermal Degradation

The cream sample and placebo sample were exposed to dry heat at 75°C for 6 hr. No degradation was observed for thermally exposed samples (75°C, 6hrs).

#### Photolytic Degradation

The cream sample and placebo samples were exposed to visible light for 240 h resulting in an overall illustration 1.2 million lux h; and UV light for 250 h resulting in an overall illustration 200 w h/m^2^ at 25 °C. Fig 5 (d) shows significant degradation for TA was also observed when the cream sample was subjected to photolytic exposure and one main degradation peak was observed at 8.448 min. Degradation for MF was also observed. All the major and minor degradation products were well-separated from the PE, MP, PP, MF, and TA peaks. The peak purity was checked for all five analytes and the results are summarized in Table 3.

**Fig. 3:**
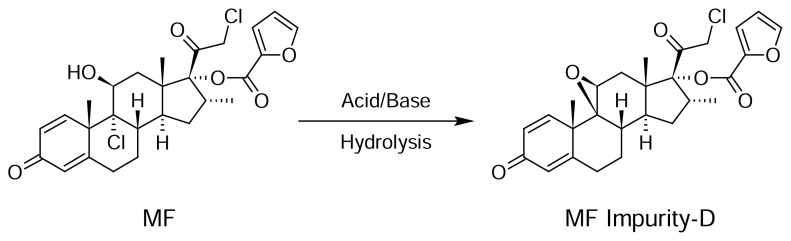
Hypothetical degradation pathway to MF Impurity-D, (9β,11β,16α)-21-chloro-16-methyl-3,20-dioxo-9,11-epoxypregna-1,4-dien-17-yl furan-2-carboxylate, from MF

**Fig. 4:**
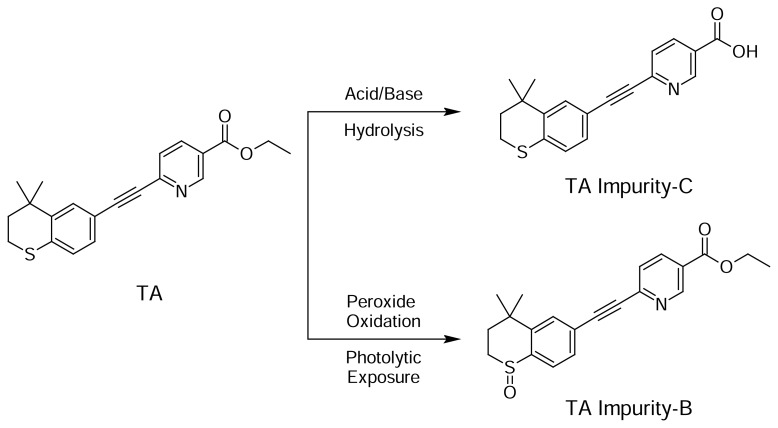
Hypothetical degradation pathway to TA Impurity-C, 6-[(4,4-dimethyl-3,4-dihydro-2*H*-thiochromen-6-yl)ethynyl]nicotinic acid, from TA by acid hydrolysis or base hydrolysis and to TA Impurity-B, ethyl 6-[(4,4-dimethyl-1-oxido-3,4-dihydro-2*H*-thiochromen-6-yl)ethynyl]pyridine-3-carboxylate, from TA by peroxide oxidation or photolytic exposure

**Fig. 5:**
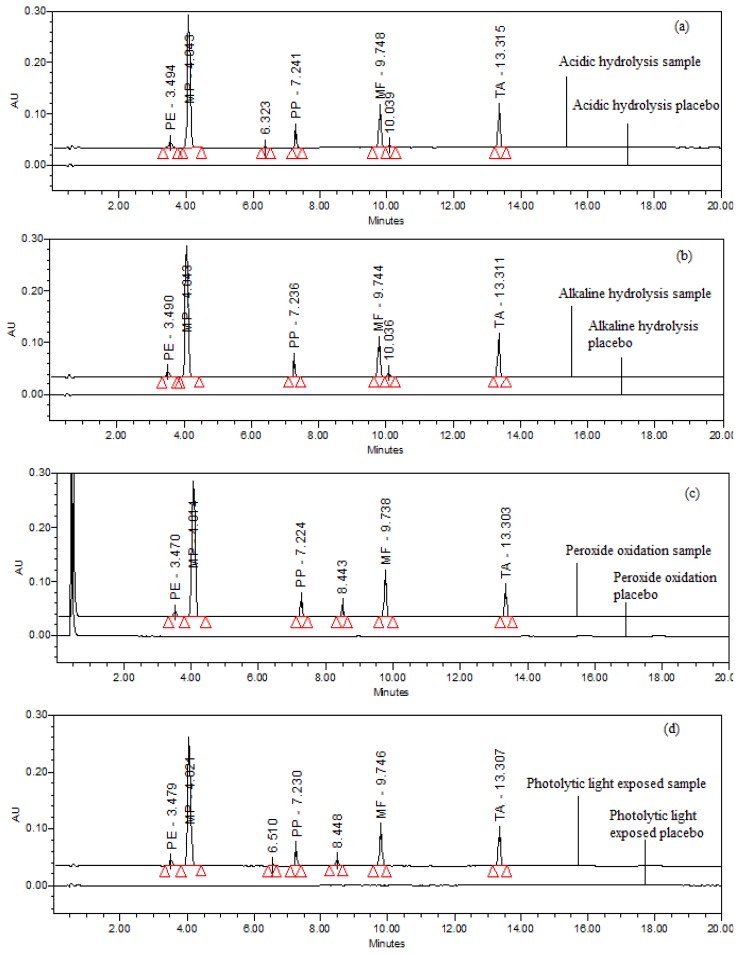
A typical overlay chromatogram of (a) acid hydrolysis sample and placebo, (b) base hydrolysis sample and placebo, (c) peroxide oxidation sample and placebo, (d) photolytic light exposed sample and placebo.

The purity and assay of PE, MP, PP, MF, and TA were unaffected by the presence of its degradation products and thus confirms the stability-indicating power of the developed method. The hypothetical degradation pathways for MF and TA [22] are presented in Fig. 3 and Fig. 4, respectively.

### Accuracy

The accuracy of an analytical method is the closeness of the test results obtained by that method compared to the true values. To confirm the accuracy of the proposed method, recovery experiments were carried out by the standard addition technique. Three different concentration levels (50%, 100%, and 150%) of standards were added to the pre-analyzed placebo samples in triplicate. The percentage recoveries of PE, MP, PP, MF, and TA at each level and each replicate were determined. The mean of the percentage recoveries (n = 3) and the % RSD were calculated. The amount recovered was within ±1% of the amount added, which indicates that the method is accurate and that there is no interference due to the excipients present in the cream sample. The results of recoveries for the assay are shown in Table 4.

### Limit of Detection (LOD) and Quantification (LOQ)

**Fig. 6:**
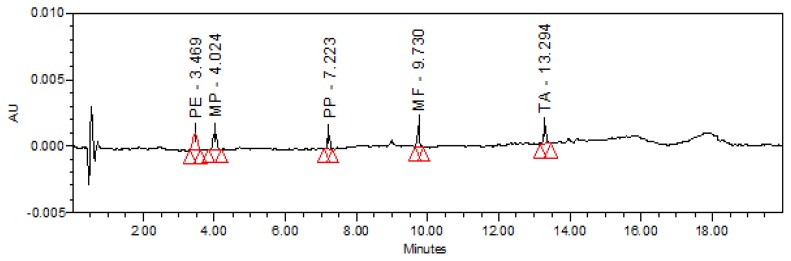
Typical chromatogram of LOQ

The LOD and LOQ were determined at a signal-to-noise ratio of 3:1 and 10:1, respectively, by injecting a series of dilute solutions with known concentrations. The limit of detection and limit of quantification values of PE, MP, PP, MF, and TA are reported in Table 5. The limit of quantification chromatogram is presented in Figure 6.

### Linearity

The linearity of an analytical method is its ability to elicit test results that are directly, or by a well-defined mathematical transformation, proportional to the concentration of the analyte. Linearity was demonstrated from the LOQ % to 150% of the standard concentration using a minimum of six calibration levels of the test concentration (LOQ-61.76 μg/mL for PE, LOQ-62.36 μg/mL for MP, LOQ-6.26 μg/mL for PP, LOQ-31.22 μg/mL for MF, and LOQ-30.45 μg/mL for TA), which gave us a good confidence on the analytical method with respect to linear range. The response was found to be linear for all PE, MP, PP, MF, and TA from the LOQ to 150% of the standard concentration. The correlation coefficient was also found to be greater than 0.9995. Bias was also found to be within ± 0.32. The result of the correlation coefficients, Y-intercept of the calibration curve, and % bias at 100% response for PE, MP, PP, MF, and TA are presented in Table 5.

### Robustness

Robustness, as a measure of the method’s capacity to remain unaffected by small, deliberate changes in chromatographic conditions, was studied by testing the influence of small changes in flow rate (1.5 ± 0.2 mL/min) and a change in the column oven temperature (50 ± 5°C). In system suitability parameters such as theoretical plates, tailing factor, and % RSD of PE, MP, PP, MF, and TA standard were studied. In all of the deliberately varied chromatographic conditions, the system suitability parameters met the acceptance criteria. Thus, the method was found to be robust with respect to the variability in the applied conditions. The results are presented in Table 1 along with the system suitability parameters of the precision and intermediate precision study. The resolution between the PE and MP peaks was observed as more than 2.4 for the robustness parameters. Thus, the method was found to be robust with respect to variability in the above conditions.

### Stability of Analytical Solutions

The stability of the sample solution was established by storage of sample solution at ambient temperature for 24 h. The cream sample solution was re-analyzed after 12- and 24-h time intervals and the assay was determined and compared against the freshly prepared standard solutions. The variability in the assay of all five substances was within ± 1% during solution stability. The results from the solution stability experiments confirmed that the sample solution was stable for up to 24 h during assay determination which are presented in Table 6.

### Filter Compatibility

Filter compatibility was performed for the nylon 0.22 μm syringe filter (Millipore) and PVDF 0.22 μm syringe filter (Millipore). To confirm the filter compatibility in the proposed method, a filtration recovery experiment was carried out by the sample filtration technique. The sample was filtered through both syringe filters and the percentage assay was determined and compared against the centrifuged sample. The sample solution did not show any significant changes in the assay percentage with respect to the centrifuged sample. Percentage assay results are presented in Table 7. The displayed result difference in % assay was not observed to be more than ±1.0, which indicates that both syringe filters have a good compatibility with sample solution.

## Experimental

### Chemicals, Reagents, and Samples

The cream sample, placebo matrix, and working standards were provided by Dr. Reddys Lab, India. HPLC grade acetonitrile and orthophosphoric acid were used (Rankem, Delhi, India). The nylon membrane filter (0.22μm), PVDF syringe filter (0.22μm), and nylon syringe filter (0.22μm) were from Millipore, Mumbai, India. Water for HPLC was generated using the Milli-Q Plus water purification system (Millipore, Milford, MA, USA).

### Equipment

The chromatographic analysis was performed using HPLC (Waters 2695 Alliance Separation Module) (Waters Milford, USA) equipped with a PDA detector, quaternary solvent manager, and autosampler system. The output signals were monitored and processed using Empower 2 software. A Cintex digital water bath was used for the hydrolysis studies. Photostability studies were carried out in photostability chamber (SUN TEST XLS+, Atlas, USA). Thermal stability studies were performed in a dry air oven (Cintex, Mumbai, India).

### Chromatographic Conditions

All chromatographic experiments were performed using the Waters X-Bridge™ C18 (50×4.6 mm, 3.5μ) column. The optimized mobile phase consisted of 0.1%v/v orthophosphoric acid in water as solvent-A and acetonitrile as solvent-B. Solvents-A and -B were filtered through a 0.22 μm nylon membrane filter and degassed under vacuum prior to use. The separation of PE, MP, PP, MF, TA, and all impurities was achieved by gradient elution using solvent-A and solvent-B. A mixture of acetonitrile and water in the ratio of 80:20 (v/v), respectively, was used as diluent. A gradient program was used as time (min)/mobile phase-A (%)/mobile phase-B (%); 0.0/90/10, 1.5/90/10, 4.5/78/22, 8/50/50, 10.5/50/50, 15/5/95, 17/90/10, 20/90/10, at a flow rate 1.5 mL/min at 50°C, detection wavelength 256 nm.

### Standard Solution Preparation

The stock solutions of PE (400 μg/mL), MP (400 μg/mL), PP (400 μg/mL), MF (200 μg/mL), and TA (200 μg/mL) were prepared by dissolving an appropriate amount of standard substances in diluent, separately. Working standard solution was prepared by mixing the above stock solutions of PE, MP, PP, MF, and TA with a final concentration of 40 μg/mL, 40 μg/mL, 4 μg/mL, 20 μg/mL, and 20 μg/mL, respectively.

### Sample Solution Preparation

An accurately weighed 1 g sample (equivalent to 1 mg of TA, 1 mg of MF) was taken into a 50 mL volumetric flask. About 35 mL of the mixture of acetonitrile and water (80:20, %v/v) was added to this volumetric flask and sonicated in an ultrasonic bath for 15 min with intermittent shaking, diluted to the volume with a mixture of acetonitrile and water (80:20, %v/v), and mixed well. A portion of the solution was filtered through a 0.22 μm nylon syringe filter and the filtrate was collected after discarding the first few milliliters.

### Placebo (Other Substances Without PE, MP, PP, MF, and TA) Solution Preparation

An accurately weighed 1 g of the placebo sample was taken into a 50 mL volumetric flask. About 35 mL mixture of acetonitrile and water (80:20, %v/v) was added to this volumetric flask and sonicated in an ultrasonic bath for 15 min with intermittent shaking, diluted to the volume with mixture of acetonitrile and water (80:20, %v/v), and mixed well. A portion of solution was filtered through 0.22 μm nylon syringe filter and the filtrate was collected after discarding first few milliliters.

## Conclusion

A gradient RP-HPLC method was successfully developed for the simultaneous determination of phenoxyethanol, methylparaben, propylparaben, mometasone furoate, and tazarotene in topical pharmaceutical dosage form. The method validation results have proven that the method is selective, precise, accurate, linear, robust, filter–compatible, and stability-indicating. Forced degradation data proved that the method is specific for the analytes and free from the interference of the placebo / known impurities / and degradation products. The run time (20.0 min) enables rapid determination of the drug. Moreover, it may be applied for the individual and simultaneous determination of phenoxyethanol, methylparaben, propylparaben, mometasone furoate, and tazarotene in the study of content uniformity, tube homogeneity, and *invitro* release test profiling of mometasone furoate and tazarotene topical pharmaceutical dosage forms, where the sample load is higher and the high throughput is essential for the faster delivery of results.

## Figures and Tables

**Tab. 1:** System suitability results (precision, intermediate precision, and robustness) for PE, MP, PP, MF, and TA

		Precision	Intermediate Precision	Flow rate 1.7 mL/min	Flow rate 1.3 mL/min	Column temp. 55°C	Column temp. 45°C
PE	N>3000	4276	3520	3794	5014	3355	3760
	T≤ 2.0	1.0	1.0	1.0	1.0	1.0	1.0
	R* ≤ 2.0	1.2	0.9	0.8	0.5	1.1	0.4

MP	N>5000	6866	5723	6366	7846	5280	5976
	T≤ 2.0	1.0	1.0	1.0	1.0	1.0	1.0
	R* ≤ 2.0	1.4	0.7	0.3	0.3	1.1	0.2

PP	N>35000	60349	57564	57290	63899	47033	52952
	T≤ 2.0	1.0	1.0	1.0	1.0	1.0	1.0
	R* ≤ 2.0	1.1	0.7	0.6	0.2	0.9	0.2

MF	N>50000	92610	78964	94801	84454	64779	66113
	T≤ 2.0	1.0	1.0	1.0	1.0	0.9	1.0
	R* ≤ 2.0	1.4	0.7	0.3	0.9	0.8	0.2

TA	N>100000	192187	179801	173149	194980	152673	166620
	T≤ 2.0	1.0	1.0	1.0	1.0	1.0	1.0
	R* ≤ 2.0	1.4	0.7	0.4	0.3	1.0	0.4

N…USP Plate count; T…USP Tailing factor; R…%Relative standard deviation; temp…Temperature;

* Determined on five values.

**Tab. 2:** Method precision and intermediate precision results

Comp.	Precision (Day-1) Analyst 1		Intermediate precision (Day-2) Analyst 2	

	% Assay#	% RSD*	% Assay#	% RSD*
PE	100.6	0.56	100.6	1.10
MP	101.4	0.33	100.4	0.90
PP	101.8	0.65	101.4	0.92
MF	101.5	0.48	101.2	1.02
TA	98.6	0.46	98.8	0.90

# …Average of six determinations;

* …Determined on six values.

**Tab. 3:** Results of forced degradation study for PE, MP, PP, MF, and TA

Comp.		Acidic hydrolysis (0.1 N HCl, RT, 45mins)	Alkaline hydrolysis (0.05 N NaOH, RT, 15mins)	Peroxide oxidation (30% H_2_O_2_, RT, 30min)	Thermal exposed (At 75°C, 6h)	Photolytic exposed (1.2 million lux h and 200 wh/m^2^)
PE	%Deg.	ND	ND	ND	ND	ND
	PA	0.195	0.222	0.757	0.191	0.213
	PTH	1.288	1.274	2.234	1.230	1.273

MP	%Deg.	ND	ND	ND	ND	5.3
	PA	0.085	0.073	1.108	0.067	0.121
	PTH	1.095	1.086	1.174	1.091	1.139

PP	%Deg.	ND	ND	ND	ND	7.7
	PA	1.188	0.916	0.751	0.661	0.766
	PTH	1.887	1.633	2.148	1.607	1.967

MF	%Deg.	8.5	12.6	ND	ND	15.1
	PA	0.339	0.666	0.289	0.285	0.486
	PTH	1.272	1.236	1.499	1.228	1.469

TA	%Deg.	3.7	1.6	33.7	ND	17.6
	PA	0.107	0.078	0.096	0.081	0.154
	PTH	1.124	1.119	1.155	1.116	1.233

ND…No Degradation; RT…Room temperature; PA…Purity angle; PTH…Purity Threshold.

**Tab. 4:** Accuracy results

Comp.		At 50%	At 100%	At 150%
PE	% Recovery #	100.0	100.0	99.9
	% R.S.D.*	0.53	0.41	0.33

MP	% Recovery #	100.3	100.3	100.2
	% R.S.D.*	0.29	0.47	0.41

PP	% Recovery #	101.5	99.9	100.0
	% R.S.D.*	0.68	0.35	0.45

MF	% Recovery #	101.0	99.8	100.1
	% R.S.D.*	0.31	0.52	0.71

TA	% Recovery #	101.0	100.2	100.5
	% R.S.D.*	0.45	0.13	0.59

* …Determined on three values;

# …Mean of three determinations.

**Tab. 5:** Evaluation of LOD, LOQ, and linearity data

Parameter	PE	MP	PP	MF	TA
LOD (μg/mL)	1.764	0.054	0.051	0.140	0.132
LOQ (μg/mL)	5.88	0.18	0.17	0.47	0.44
Linearity range (μg/mL)	5.88–61.76	0.18–62.36	0.17–6.26	0.47–31.22	0.44–30.45
Correlation coefficient	0.9997	0.9999	0.9999	0.9999	0.9999
Intercept (a)	216.992	276.567	−349.533	90.771	−569.537
Slope (b)	1730.336	39762.69576	35039.13	17737.78	16789.99
Bias at 100% response	0.307	0.017	−0.241	0.025	−0.168

**Tab. 6:** Solution stability results

% Assay	Initial	After 12 hrs.	After 24 hrs.
PE	100.6	100.1	100.5
MP	101.4	101.2	100.6
PP	101.8	101.7	100.9
MF	101.5	101.9	101.4
TA	98.6	98.5	98.0

**Tab. 7:** Filter compatibility results

% Assay	Centrifuged Sample	PVDF filter 0.2μm	Nylon filter 0.2μm
PE	98.6	98.5	99.2
MP	101.2	100.7	101.6
PP	101.9	101.2	101.8
MF	101.9	101.3	101.5
TA	98.5	98.0	98.7
